# TAPP - Stuttgart technique and result of a large single center series

**DOI:** 10.4103/0972-9941.27730

**Published:** 2006-09

**Authors:** R Bittner, B J Leibl, C Jäger, B Kraft, M Ulrich, J Schwarz

**Affiliations:** Department for General and Visceral Surgery, Marienhospital Stuttgart, Boeheimstrasse 37, D-70199 Stuttgart

**Keywords:** Hernia repair, laparoscopy, TAPP

## Abstract

Laparoscopic hernioplasty is assessed as a difficult operation. Operative technique determines the frequency of complications, the time of recovery and the rate of recurrences. A proper technique is absolutely necessary to achieve results that are superior to open hernia surgery.

**Technique:**

The key points in our technique are 1) use of nondisposable instruments; 2) use of blunt trocars, consisting of expanding and non-incisive cone-shaped tips; 3) spacious and curved opening to the peritoneum, high above all possible hernia openings; 4) meticulous dissection of the entire pelvic floor; 5) complete reduction of the hernial sac; 6) wide parietalization of the peritoneal sac, at least down to the mid of psoas muscle; 7) implantation of a large mesh, at least 10 cm × 15 cm; 8) fixation of the mesh by clip to Cooper's ligament, to the rectus muscle and lateral to the epigastric vessels, high above the ileopubic tract; 9) the use of glue allows fixation also to the latero-caudial region; and 10) closure of the peritoneum by running suture.

**Results:**

With this technique in 12,678 hernia repairs, the following results could be achieved: operating time - 40 min; morbidity - 2.9%; recurrence rate - 0.7%; disability of work - 14 days. In all types of hernias (recurrence after previous open surgery, recurrence after previous preperitoneal operation, scrotal hernia, hernia in patients after transabdominal prostate resection), similar results could be achieved.

**Summary:**

Laparoscopic hernia repair can be performed successfully in clinical practice even by surgeons in training. Precondition for the success is a strictly standardized operative technique and a well-structured educational program.

Among all inguinal hernia repair techniques that utilize a preperitoneal placement of mesh, the advantage of laparoscopic hernioplasty (TAPP) is based on the fact that any type of groin hernia can be treated safely and effectively in a standardized way.[1] Diagnostic laparoscopy allows an immediate evaluation of the type of hernia on both the sides. In case of difficult preparation of a large hernial sac, it is possible to observe the sac and its content continuously. A disadvantage of the TAPP is that the placement of the mesh cannot be checked while deflating the pneumoperitoneum. The mesh needs to be placed without any wrinkles, with a wide overlap of the hernia defect (>3–5 cm), whereas the parietalization plays the most important role.

The degree of difficulty of TAPP is not only determined by pathologic-anatomic substrate of hernia (type, size, cicatrization, associated lipoma) but also by physical attributes of the patient (size; weight; pelvic width; local obesity; strength of musculature of the abdominal wall; thickness and distensibility of the abdominal wall; abdominal previous surgery, e.g., appendectomy).

To perform a first TAPP, the ideal patient is older than 60 years, has a flabby abdominal wall, has a poor muscular tone, has a wide pelvis, is slightly obese, has a small direct hernia and has had no previous abdominal surgery.

## Standard technique of transabdominal preperitoneal hernia repair (TAPP)

The patient lies supine and flat on the operating table with both arms placed by the side. After obtaining the pneumoperitoneum, the patient needs to be placed in a Trendelenburg position and turned at an angle of 10–20° towards the surgeon, so that the surgeon can approach the inguinal region without the hindrance of the intestinal loops.

To place the optical trocar, an incision of 1 cm needs to be made longitudinally superior of the umbilicus down to the umbilical base. The opposite wound edges are grasped using strong Backhaus clamps to lift up the abdominal wall. Thus, Veress needle can be positioned to induce a pneumoperitoneum. Semm's safety tests are always performed to ensure correct intraperitoneal position of the needle. During insufflation, the intra-abdominal pressure and the gas flow need to be observed. To ensure correct position of the tip of the Veress-needle in the abdominal cavity, the pressure should be low (about 0 mm/Hg), whereas the flow is required to be adequately high (about 2 L/min). Then insufflation can be continued until a maximal pressure of 12 mm/Hg is reached. The optical trocar is then placed. Again, the abdominal wall should be held tense by using Backhaus clamps. The optical trocar is screwed into the abdominal cavity softly, and the optic can be inserted. Usually we use a 30° angle optic (Karl Storz GmbH, Germany).

It is customary to perform a diagnostic laparoscopy to inspect the upper abdomen, including the liver. To expose the inguinal area, the patient needs to be positioned as mentioned above. The surgeon should stand on the opposite side of the hernia. The camera operator is required to sit on the hernia side. The video tower is placed at the feet of the patient, whereas the scrub nurse stays on the left side.

In case of bilateral hernia, all the trocars are placed at the umbilical and midclavicular levels (right 12 mm, left 5 mm). In case of a unilateral hernia, one trocar can be positioned above the umbilicus at the hernia side, whereas the contralateral trocar may be placed below the umbilicus to avoid collision with the optical trocar.

In order to avoid any injury to intra-abdominal organs, trocars should always be inserted under vision. Without exception, blunt and reusable trocars with expanding and non-incisive cone-shaped tips are used (Karl Storz GmbH, Germany). Consequently, injuries of epigastric vessels causing bleeding complications as well as major tissue trauma, followed by late postoperative hernia formation, can be avoided completely.

The dissection of the right inguinal area begins with a curved and a spacious opening of the peritoneum, starting in the region of the anterior superior iliac spine, going to the myopectinal orifice and ending at the medial umbilical ligament. In case of a prominent fatty ligament which obscures the access to the inguinal region, it should not be cut (caveat: bleeding from a non-obliterated umbilical artery may ensue), but the incision of the peritoneum should be enlarged towards the cranium in a ‘J’ shape.

The dissection of the inguinal region is done in accordance with a strict concept. Before dissecting the myopectinal orifice with the hernial sac, a preparation of the lateral and medial compartments is required, particularly in an obese patient. It is advantageous to identify important landmarks – such as the rectus muscle and symphysis, as well as testicular vessels laterally. Consequently, risk of injury to the spermatic cord, iliac vessels, nerves and urinary bladder can be eliminated. Dissection of the hernial sac should not be performed before demonstrating both the compartments clearly. An early identification of the epigastric vessels is recommended. In addition, it is advisable to free the epigastric vessels of fat so as to uncover the inner inguinal ring exactly. In case of a lipoma at the entry into the inguinal canal, dissection is required to identify to the hernial sac.

By following a so-called ‘cobweb-like nonvascular zone,’ the space of Retzius (medial compartment) as well as the Bogros’ space (lateral compartment) can be dissected in a blunt manner. The left hand is used to pull firmly on the leaf-like opened peritoneum, while the right hand performs either a blunt or a sharp dissection with a Metzenbaum scissors, which is connected to unipolar diathermy. As a matter of principle, accurate hemostasis is required to identify landmarks and obtain excellent exposure.

Dissecting a direct hernia is a simple process: immediately after dissecting the medial compartment a preperitoneal lipomatous tissue is observed at the direct hernial orifice. By placing the peritoneum as well as the lipomatous tissue on tension, the transversalis fascia (which forms the hernial sac) becomes visible and appears to be a white circular structure. Stepwise, the lipomatous tissue gets separated from the transverse fascia without penetrating it. Furthermore, a thorough hemostasis with the use of monopolar diathermy as well as a meticulous dissection of the hernial sac play an important role in reducing postoperative formation of serohematoma. It has been observed that as the dissection progresses medial, Corona mortis is observed to occur in 20% of patients. Again, careful attention is required to avoid bleeding.

The femoral hernial orifice is located in an angle formed by the Cooper's ligament (iliopubic tract inserting into the pubic os) and the iliac vein. Thus, the femoral hernial orifice can be exposed. Due to the proximity to the femoral vein, the dissection needs to be performed very carefully.

Dissection of the indirect hernial sac is much more difficult as compared to the direct hernia, especially when the sac is long and contains scar formation near the cremaster encircling the spermatic cord. Proceeding systematically, at first the testicular vessels, located caudal and lateral, should be dissected. Afterwards, the hernial sac is separated off the adhesions to the above-mentioned structures by starting from caudal lateral and then going to cranial medial. Under careful hemostasis, dissection is achieved partly bluntly and partly sharply. Especially in large hernias, the double-instrument-rope-ladder method should be used, which means that the scissors in the right hand are replaced by a second forceps. Now, the hernial sac can be dissected step by step off the inguinal canal by adopting the rope-ladder principle. To release and separate the hernial sac off the spermatic cord, a fine and superficial coagulation of the adhesions normally suffices.

Quite often, strong adhesions can be found at the entrance of the inguinal canal between the hernial sac and cremaster muscle, as well as the medial edge of the epigastric vessels. Again, superficial coagulation suffices to bluntly peel off the hernial sac. A stepwise progress under permanent view of the vessels of the spermatic cord permits the surgeon to reach the tip of the hernial sac. The following procedure is simple and only the freeing up of the vas deferens is left. Once again, the procedure is similar to the one used for the testicular vessels. This time, the dissection goes from cranial lateral to caudal medial. The usage of scissors combined with monopolar diathermy allows even firm adhesions to be dissected free. (Caveat: Dissect a safe distance away from the vas deferens!)

The final step of dissecting the groin includes parietalization, at which peritoneum is dissected off the spermatic cord and the spermatic fascia beyond the middle region of the psoas muscle. In doing so, even the flimsiest of the connections between the peritoneum and the retroperitoneal space and spermatic fascia and spermatic cord respectively should be disconnected. The purpose of parietalization is to prevent the mesh (placed over the hernial orifices) from being lifted up by the remaining connective tissue during peritoneal closure; this may especially occur laterally. Thus, a later recurrence of the hernia from caudal and lateral caused by shifting of the fatty tissue is prevented. One should be able to lift the peritoneum without causing movement of the mesh. Now the preparation of the groin is completed.

During the next step, by using a forceps, the 10 cm × 15 cm mesh is pushed through the 12 mm trocar and placed in an umbrella-like manner in the dissected area. The myopectinal orifice should be covered adequately without allowing any folds in the mesh. By using two clips (Endopath Hernia Stapler, Ethicon), the mesh should be fixed to the symphysis and Cooper's ligament. Two clips are placed on the rectus muscle medial to the epigastric vessels and two clips at the transverse fascia located lateral to the epigastric vessels. Thereby, the distance between clips and ileopubic tract should be at least 2 cm. Placing clips below the iliopubic tract and 1–2 cm above is strictly avoided because doing so could lead to injuring large vessels (triangle of doom) or nerves (triangle of pain). To prevent shifting during surgery, fixation of the mesh could also be done by using sutures or fibrin glue (Tissocol, Baxter). It is advisable to use a larger mesh (12 × 17 cm) for major - defects (measuring more than 5 cm).

As soon as the mesh is placed in position, the peritoneal incision should be approximated using an absorbable, industrially manufactured suture, whose ends get fixed with absorbable clips (Lahodny-Suture, Ethicon). At the time of suturing the peritoneum, the intra-abdominal pressure is reduced to 6–8 mmHg, thereby allowing a tension-free peritoneal closure. An alternative is to close the peritoneum with a simple continuous suture with intracorporeal knotting.

The procedure is terminated by removing all trocars under vision. In case bleeding is observed from the port sites, it is possible to control it by electrocoagulating the area with a grasper from the contralateral port. Finally, after deflating the abdominal cavity, the optical trocar needs to be removed. Therefore, an accurate closure of the fascia with a strong suture is required; because in contrast to the lateral working trocars, the fascial opening at the optical trocar is not covered by muscles.

## MATERIALS AND METHODS

Between April 1993 and December 2005, we have performed a total of 12,678 hernia repairs using the TAPP technique. The patient population was completely unselected, and all types of hernias were included [Table 1]. The percentage of conventional hernia repair was less than 2%. All patients were included in a follow-up program with clinical examinations at 4 weeks, 1 year and 5 years after the operation. All patients’ data were collected prospectively and was entered into a database. More than 90% of the patients were seen at least once during the follow-up period.

**Table 1:** Types of hernia (N = 12687)

		%
II (indirect)	3696	24.1
IIIa (direct)	4571	32.6
IIIb (indirect/combined)	2634	25.9
IIIc (femoral)	337	3.2
IV (recurrrent)	1725	14.2

## RESULTS

With strict application of our technique in 12,678 laparoscopic hernia repairs (TAPP), the following results have been achieved: operation time 40 min (12–276), morbidity 2.9%, reoperation rate 0.44%, recurrence rate 0.68%, period of disability 14 days (1–100). Tables 2, 3 and 4 show the frequency of intraoperative, early postoperative and late postoperative complications. Most of the complications were seen in the learning curve and in complicated hernias [Table 5]. Tables 6, 7 and 8 show that with experience, TAPP can provide excellent results – even in the most complicated hernias.

**Table 2:** Intraoperative complications [N=108 (0,85%)]

Injury to lateral femoral cutaneous nerve	N = 33 (0.26)
Bleeding (Trocar, inguinal)	N = 42 (0.33)
Urinary bladder injury	N = 10 (0.08)
Bowel injury	N = 11 (0.09)
Injury to vas deferens	N = 2 (0.02)
Other	N = 8 (0.06)

Figures in parentheses are in percentage

**Table 3:** Postoperative complications [N = 156 (1.23%)]

Complication	N (%)
Urinary retention	55 (0.43)
Bleeding	34 (0.26)
Epididymitis / orchitis	9 (0.016)
Mesh infection	10 (0.09)
Urinary tract infection	8 (0.07)
Seroma (Reoperation)	14 (0.09)
Thrombosis	6 (0.04)
Allergic reaction	5 (0.04)
Wound infection	6 (0.04)
Ileus	3 (0.02)
Pulmonary embolism	2 (0.01)
Apoplexy	1 (0.01)
Myocardial infarction	1 (0.01)
Bowel atony	2 (0.01)

**Table 4:** Long term complications [N=92 (0.72%)]

Complication	N (%)
Trocar hernia	68 (0.53)
Testicular atrophy	6 (0.05)
Seroma (persistent)	6 (0.05)
Chron. pain (reoperation)	6 (0.05)
Other	3 (0.02)
Ileus (reoperation)	3 (0.02)

**Table 5:** Results of TAPP

Operations	Primary hernia (N= 10962)	TAPP after anterior repair (N=1590)
Operative time (Median, minutes)	40	45
Morbidity	2.8%	3.5%
Reoperation rates	0.3%	0.6%
Recurrence rates	0.7%	1.0%
Disability (median, days)	14	17
Age (median)	17–97 (59)	18–92 (61)
BMI (median)	25	25

TAPP -Transabdominal preperitoneal repair

**Table 6:** Scrotal hernia – learning curve (N=702)

Operations	1–235	236–470	470–702
Time interval	7/’93–6/’98	6/’98–5/’01	5/’01–12/’05
Operative time (median, minutes)	65	65	60
Morbidity	5.9%	5.5%	1.7%
Reoperation rates	1.7%	0.8%	0.8%
Recurrence rates	4.6%	1.3%	0.86%
Disability (median, days)	21	14	12

**Table 7:** TAPP in complicated hernia - after transabdominal prostate resection (N=236)

Operations	1–80	81–160	161–236
Time interval	3/’94–12/’99	12/’99–4/’03	4/’03–12/’05
Operative time (median, minutes)	50	51	54
Morbidity	8.7%	7.5%	2.6%
Reoperation rates	1.25%	1.25%	2.2%
Recurrence rates	1.25%	1.25%	-
Disability (median, days)	21	14	14

TAPP - Transabdominal preperitoneal repair

**Table 8:** TAPP after preperitoneal mesh repair (N=135)

Operations	1–45	46–90	91–135
Time interval	6/’93–12/’98	12/’98–2/’02	2/’02–11/’05
Operative time (median, minutes)	82.5	72	77
Morbidity	14%	8%	2%
Reoperation rates	2.2%	2.2%	2.2%
Recurrence rates	-	-	2.2%
Disability (median, days)	18	17	17

TAPP - Transabdominal preperitoneal repair

## CONCLUSION

Laparoscopic hernia repair by the TAPP technique is an excellent operation for treatment of inguinal hernia. In comparison to open surgery, morbidity and recurrence rate are favorably low. Precondition for excellent results is the strict application of a standardized technique. In experienced hands, all types of hernias, including large scrotal hernias and recurrent hernias after previous preperitoneal repair, can be operated with low morbidity and recurrence rates. The same is true for hernias in patients with a history of previous preperitoneal surgery, e.g., transabdominal prostate resections. Evidence-based data show that laparoscopic hernia repair is significantly better than the conventional operations, especially with respect to all pain-associated parameters.[2,3] However, to achieve favorable results, a strong educational program in laparoscopy is highly recommended.
